# Epidemiological profile of long-term leave for psychiatric illnesses

**DOI:** 10.1192/j.eurpsy.2024.1184

**Published:** 2024-08-27

**Authors:** S. Chemingui, M. Mersni, M. Bani, H. Ben Said, H. Khiari, I. Youssef, N. Mechergui, D. Brahim, G. Bahri, I. Yaich, C. Ben Said, N. Bram, N. Ladhari

**Affiliations:** ^1^Occupational pathology and fitness for work department Charles Nicolle Hospital, tunis, Tunisia; ^2^Occupational pathology and fitness for work department, Charles Nicolle Hospital; ^3^Forensic Psychiatry department, Razi Hospital, La Manouba, Tunis, Tunisia

## Abstract

**Introduction:**

Long-term leave for psychiatric illness is the most frequently prescribed reason for leave, and appears to be on the increase in recent years.

**Objectives:**

To draw up a sociodemographic, occupational and clinical profile of workers who have taken long-term sick leave for psychiatric illness

**Methods:**

Retrospective descriptive study involving the medical files of workers from both the public and private sectors, having benefited from long-term sick leave over a period going from August 17, 2022 to September 12, 2023, referred to the occupational medicine and pathology department of Charles Nicolle Hospital in Tunis for medical fitness-for-work assessment. Data collection was based on a pre-established synoptic form.

**Results:**

During the study period, we identified 639 long-term sick leave prescribed for psychiatric illnesses. Our study population was predominantly female, with a sex ratio of 0.29 and a mean age of 46.82 ± 25.06 years. Sixty percent of employees were married. The most represented occupational category was nurses (33%). Average job seniority was 17.21±10.41 years. Depressive syndrome was the most common psychiatric pathology in our population (80.3%), followed by bipolar disorder (6.4%) and anxiety disorder (5%). Long-term sick leave was prescribed by a psychiatrist working in the private sector in 90.3% of cases. The average duration of leave was 63.70±31.58 days. The triggering factor was work-related and social in 33.6% and 30.1% of cases respectively. The agents returned to work after the long-term sick leave in 92% of cases.

**Conclusions:**

Long-term sick leave for psychiatric reasons is a handicap to productivity in society. Non-occupational factors are thought to be responsible for these mental health disorders. Setting up and improving social structures in the workplace would reduce the number of cases of long-term sick leave

**Disclosure of Interest:**

None Declared

